# Controlling Parasitic Nucleation in Methylamine‐Treated Perovskite Films via Artificial Seeding and Phase‐Field Simulations

**DOI:** 10.1002/smsc.70301

**Published:** 2026-05-15

**Authors:** Emilia R. Schütz, Martin Majewski, Olivier J. J. Ronsin, Jens Harting, Lukas Schmidt‐Mende

**Affiliations:** ^1^ Department of Physics University of Konstanz Konstanz Germany; ^2^ Helmholtz Institute Erlangen‐Nürnberg for Renewable Energy (HIERN) Forschungszentrum Jülich GmbH Erlangen‐Nürnberg Germany; ^3^ Department of Chemical and Biological Engineering and Department of Physics Friedrich‐Alexander‐Universität Erlangen‐Nürnberg Nürnberg Germany

**Keywords:** analytical model, methylamine treatment, phase‐field simulation, seeded growth

## Abstract

Large perovskite crystals with reduced defect density enable superior charge transport and stability. Therefore, controlling their nucleation and growth is key to advancing high‐performance optoelectronic devices based on perovskite semiconductors. Through the combination of a methylamine treatment and artificial nucleation sites, we demonstrate for the first time the preparation of hundreds‐of‐micrometer large perovskite crystals in a predefined spatial pattern. Nonetheless, certain configurations may lead to unwanted parasitic nucleation. To predict and mitigate this effect, we employ phase‐field simulations and develop an analytical model. Their predictive capability is demonstrated across three distinct material–substrate systems, enabling precise control over nucleation and subsequent crystal growth. Notably, the only material‐specific input required is the nucleation density (i.e., the number of crystals nucleated per unit area on an unpatterned substrate). This generality makes the models broadly applicable to diverse material systems for achieving controlled 2D crystallization for improved optoelectronic device performance.

## Graphical abstract

Designed crystal nucleation can be achieved by seeded growth. The occurrence of parasitic nucleation can be predicted by modeling. Unmatched control over thin‐film crystallization is achieved over a variety of perovskite‐material‐substrate combinations.

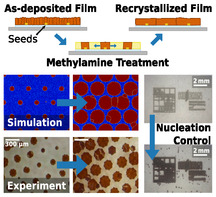

## 1 Introduction

Perovskite materials attracted significant attention in recent years due to their remarkable optoelectronic properties [1]. The formation of large perovskite crystals, ideally to single‐crystalline devices, is considered highly desirable, as it reduces defects at grain boundaries [2] and enhances the stability of the material [3]. These characteristics are crucial for improving the performance and longevity of perovskite‐based devices.

An effective method to achieve large grain sizes in perovskite films is through methylamine treatment. Through the reaction between methylamine gas and the perovskite material, an existing film can be dissolved and recrystallized with altered properties that are determined only by this single treatment step [4]. As the original film quality is not decisive for the final film properties, this treatment potentially eliminates the need for meticulous optimization of earlier steps. In addition, it enables straightforward upscaling by facilitating large‐scale fabrication without demanding high precision in the initial film deposition. Crucially, the method can be used to slow down a nucleation and growth process, producing exceptionally large grains that are beneficial for applications in solar cells [5, 6] and photodetectors [7].

The ability to pattern substrates with artificial nuclei offers further control over the crystallization process, allowing for the predetermination of grain size, position, and orientation [8, 9]. This level of control could be particularly advantageous for applications requiring high crystallinity and precise structural organization, such as in LEDs, laser devices, and lateral perovskite devices such as back‐contacted solar cells. Moreover, patterned growth enables us to create interesting model systems to deepen our understanding of the crystallization process itself.

Controlled 2D growth has been explored in various material systems, and its broader implications extend beyond perovskites [10, 11, 12, 13, 14]. The ability to grow highly crystalline elements directly on circuit structures with controlled properties opens up possibilities for numerous technological applications. However, despite its potential, there has been limited research into understanding and simulating these types of growth processes. Much of the current knowledge is based on trial and error, underscoring the need for a more systematic investigation into the limitations and capabilities of patterned growth techniques.

To go beyond trial and error, a theoretical description of the underlying processes is required. Phase‐field (PF) simulations offer an attractive approach to this problem, since they can numerically describe the phase change from amorphous to solid [15, 16, 17]. PF models are coarse‐grained models with diffuse interfaces, based on the minimization of a free energy. The phenomenon of nucleation can be included in PF models by applying fluctuations on the evolution equation [18]. By including this effect, heterogeneous nucleation and crystal growth in confined spaces have been investigated [19]. The solution processing of perovskite systems has been investigated by studying the interplay of solvent evaporation and crystal growth [20] and furthermore including nucleation [21, 22]. Nevertheless, to the best of our knowledge, the presence of seed crystals in the initial state and allowing additionally for spontaneous nucleation has not been investigated in the literature so far.

In this work, two of these models, a phase field simulation and an analytical model, are used to optimize the experimental patterning of a methylamine‐treated perovskite thin film. The main problem with patterning is the appearance of “parasitic” crystals at spots which are not intended by the patterning. We recover this behavior with PF simulations [23], where we place crystals in the initial state in an hexagonal lattice and allow for additional nucleation. Based on the simulations, we propose an analytical model describing this behavior. Our models show that the main criteria for the appearance of parasitic crystals is an dimensionless parameter, calculated from the distance between the seeds and the nucleation density. Consequently, we predict this distance depending only on the nucleation density of the system. Hence, we are able to evaluate the feasibility of any arrangement of seeds to fully control the nucleation and growth process and avoid parasitic crystallization.

The study is structured as follows: First, we introduce the methylamine treatment and the patterning processes. Then, we describe the theoretical results including the phase‐field simulations, the derivation of the analytical model, and the validation of the predictions for three different experimental systems. Finally, the generality of the models is discussed before we conclude with a summary of our finding.

## 2 Methylamine Treatment Process

The methylamine post‐treatment we apply in this work is a versatile instrument in modifying perovksite thin films [4] and achieving up to millimeter‐sized grains [5, 7]. It allows us to achieve controlled, very slow nucleation and crystallization, yielding a compelling model system to observe growth mechanisms.

**FIGURE 1:**
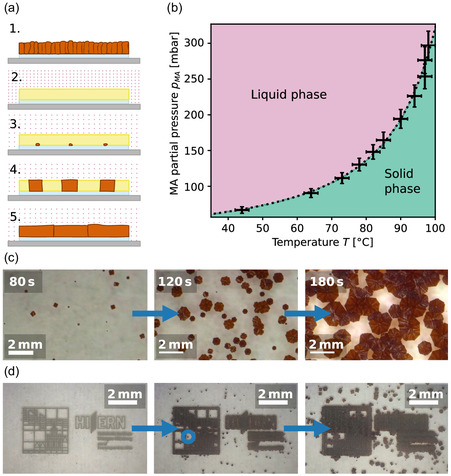
(a) Schematic of the methylamine treatment process. (b) Phase diagram of the methylamine process: For sufficiently high methylamine (MA) partial pressures *p*_MA_ at sufficiently low temperatures *T*, the system enters a liquid intermediate state. Through a reduction in *p*_MA_, the system is brought just across the phase boundary, where slow nucleation and recrystallization ensues. The quantitative values presented here were derived for triple cation perovskite on an ITO substrate. (c) Images of a sample during recrystallization. (d) Images of the successful artificial nucleation of the perovskite. Note that the underlying Au structure is not visible, only the resulting perovskite crystals. Where the nucleating structures are too far apart, additional “parasitic” nuclei grow (an example is circled in blue).

The full treatment process is shown in Figure 1a. It is based on the reaction between methylamine gas and the perovskite lattice: The amine replaces the Pb‐I bonds due to its proclivity to coordinate Pb^2+^ [24]. Consequently, the perovskite lattice collapses into a liquid intermediate state, with the methylamine acting essentially as a gas‐phase solvent [25]:

(1) $${\mathrm{ABX}}_{3(s)}+x{\mathrm{CH}}_{3}{{\mathrm{NH}}_{2}}_{\left(g\right)}⇌{\mathrm{ABX}}_{3}\cdot x{\mathrm{CH}}_{3}{\mathrm{NH}}_{2(l)}$$

Once the methylamine leaves the film, inducing a state of supersaturation, the perovskite phase recrystallizes. In Figure 1b, the phase diagram of this system is shown. Experimentally, we achieve liquefaction or recrystallization by changing the system temperature *T* and the methylamine partial pressure *p*_MA_. Figure 1c shows the nucleation and recrystallization process, as observed in situ. Grains nucleate at random points, then grow outward until they cover the entire surface. For a more detailed explanation of the treatment process, see [7].

During this process, the exact grain location, i.e., the points of nucleation, remain functionally random, as they are likely to be determined by impurities or protrusions on the substrate. To gain complete control over the final film, we thus introduce artificial seeds onto the substrate.

### 2.1 Gold Patterning for Nucleation Control

Generally, nucleation most likely occurs heterogeneously at the substrate/liquid interface, and the likelihood of nucleation is highly sensitive to the substrate material. Gold has been found to be an effective seed material for various substances [26, 27], including perovskites [8]. By using lithographically patterned gold dots as nucleation centers, we can thus create preferred points for nucleation on our substrate. By carefully selecting the recrystallization parameters, the temperature *T*, and the methylamine partial pressure *p*_MA_, we maintain a state of supersaturation that prevents nucleation at the substrate interface but allows for it at the gold sites. This enables us to effectively control the precise arrangement of grains in the film. For a more detailed explanation of the nucleation control mechanism, see the Section S2. In Figure 1d, the controlled nucleation and growth on the gold patterned substrate is shown.

While this scheme delivers the controlled growth of large‐grained perovskite structures, there are limits to the seed pitch at which this process works well: As there is a remaining nucleation probability at the substrate interface, parasitic nucleation is observed between the artificial seeds above a certain seed distance *D*_max_ (see the features circled in blue in Figure 1d). The extent to which unwanted, parasitic, grains appear is of great importance for future applications: *D*_max_ effectively limits the size of any circuit or device into which the grown grains are to be integrated. This question does not only pertain to this specific material system, but to all 2D nucleation systems, in some of which parasitic grain formation is also clearly visible [10]. In the following, we approach this problem from a theoretical perspective by using phase‐field simulations and constructing an analytical model.

## 3 Results

### 3.1 Phase‐Field Predictions for the Maximal Seeding Distance

In this chapter, phase‐field (PF) simulations are presented. The equations governing the simulation have been published [23] and are described in the Section S3. The simulation parameters are shown in Table S1. Simulations are performed in a 2D top view with periodic boundary conditions in both directions. The initial amorphous material transforms to the crystalline phase with the system’s evolution described using an order parameter. To allow for handling multiple crystals, each crystal is uniquely labeled with a marker field. Nucleation is triggered by thermal fluctuations of the order parameter.

To mimic the gold patterning of the substrate, the initial state of the simulation features round crystals positioned in a hexagonal lattice. Like in the experiments, we vary the distance between the seed crystals. The crystals grow in a pure reservoir with a constant interface velocity, while additional crystals may nucleate, depending on the initial conditions and the random fluctuations. Ten different simulations are performed for each pitch, and the number of parasitic grains is recorded.

The resulting number of parasitic crystals depends on the nucleation rate *κ* and the growth rate of the nucleated crystals *v*_g_. These quantities depend on the combination of material and substrate. However, they are often unknown and difficult to measure experimentally. To connect the simulation results to experimental systems, we scale the results to the final crystal density *η* on an unpatterned substrate:

(2) $$\eta =\frac{\text{Number of crystals}}{\text{Area}}$$

*η* depends on crystal surface tension *κ* and crystal growth speed *v*_g_, and is (relatively) easily accessible experimentally. The final results depend only on this single material property and the pitch *D*, which allows for the comparison between simulation and experiments.

**FIGURE 2:**
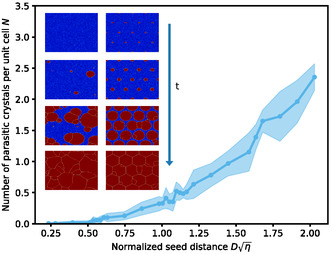
The average number of parasitic crystals in dependence on the pitch D. The shaded area depicts the standard deviation over 10 simulation runs. Note that the only difference between the simulations, for the same pitch, is the random fluctuations. Insets: Snapshots of the time evolution of a substrate without (left) or with (right) initial seed crystals in the simulation. The amorphous phase is depicted in blue, and the crystalline phase is shown in red.

The number of parasitic crystals *N*, averaged over 10 simulation runs and scaled by the number of initial seeds, is shown in Figure 2. As described above, the pitch is scaled with the square root of the nucleation density *η*. We observe that there is a range of small pitches, up to $D\sqrt{\eta }$= 0.5, where (nearly) no parasitic nucleation occurs. Interestingly, exactly no parasitic nucleation occurs only for smaller values up to $D\sqrt{\eta }$= 0.25. Above $D\sqrt{\eta }$= 0.5, the number of parasitic grains increases. The results are robust against changes in the sizes of the seed crystals and lattice arrangement (see Sections S2.5 and S2.6). To gain a more fundamental insight into the number of parasitic grains, we develop an analytical model of the process in the following section.

### 3.2 Analytical Model

**FIGURE 3:**
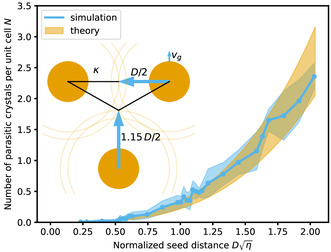
The number of parasitic crystals obtained from the simulation and calculated analytically (Equation (4)). Inset: sketch visualizing the parameters of the analytical model. The three crystals (orange) represent a unit cell of the hexagonal lattice. The crystals grow with a growth speed of *v*_g_, while additional crystals nucleate in the amorphous phase with a rate of *κ*. The arrows and shaded circles visualize the lower and upper integration limits of the model (see main text).

In order to derive the model, we consider a single triangle of crystals. This represents the basic unit of the hexagonal lattice used in the experiments. The appearing crystals have an initial size of *r*_0_ = 0 and growth with a speed of *v*_g_. An amorphous precursor phase is present between the crystals. In the amorphous phase, the nucleation rate is *κ*, which is assumed to be constant in time and space. The number of parasitic grains *N* can be calculated by

(3) $$N=\int_{0}^{\infty }\kappa A(t)$$

where *A*(*t*) is the surface which is not crystallized at time *t*. When crystals are placed initially, *A*(*t*) has to account for the growth of these crystals. For simplicity, we neglect the effect of parasitic crystals on the available volume for crystallization. This will lead to an overestimation of crystals for large seed distances. We expect to have a nucleation induction time *t*_0_, which is the time between the start of crystal growth and the first nucleation event. Since the crystal growth of the seeds will result in full coverage of the available space within a finite time, the upper integration limit can be set to a finite value. This results in (derivation in the section S4)

(4) $$N=\frac{\kappa I^{3}}{4v_{g}}\left(1-\frac{\pi }{12}\right)+\frac{\kappa I^{2}t_{0}}{2}\left(\frac{t_{0}^{2}v_{g}^{2}}{3}-1\right)$$

where *I* is a parameter in the integration limit. A lower limit for the number of parasitic crystals can be set, if *I* = *D*. At this point, the initial crystals will first touch, but do not cover the entire space. An upper limit can be determined by using the time it takes for the seed crystals to cover the entire available space, *I* = 1.15*D*. In this case, parts of the system are considered twice, resulting in an overestimation of the number of crystals. The limits are sketched in the inset in Figure 3. *v*_g_, *κ*, *t*_0_, and *η* can be measured from a PF simulation of nucleation on a bare substrate (see Section S4.1).

In principle, *η* could be calculated from the nucleation rate *κ* and the growth rate of the nucleated crystals *v*_g_. However, nucleation models are not precise enough to estimate the crystal density (see Section S4.2). Therefore, *η* is extracted from the simulations.

The results of the analytical model are plotted on top of the simulation results in Figure 3. The two curves overlap. Note that with this set of parameters, the impact of the nucleation induction time is comparatively small.

### 3.3 Experimental Verification for Different Nucleation Densities

Both model and simulation are capable of describing the problem at hand with remarkable generality; the only system specific parameter to the material system is the nucleation density *η* observed on a substrate without seeds. To validate the theoretical results, we therefore choose different model systems which feature different values of *η*.

Within the experimental setup described in Section 2, there are several ways to modify *η*. A possible approach is to vary the atmospheric treatment conditions *T* and *p*_MA_: By having the system recrystallize in a more or less pronounced state of supersaturation, the nucleation probability, and thus density, can be shifted substantially. The closer to the phase boundary the system lies in the end, the smaller the nucleation rate, and hence the nucleation density.

Another approach is to vary the material system: The specific substrate/perovskite combination determines the interfacial energy in the liquefied state, thus changing the likelyhood of heterogeneous nucleation. In addition, impurities and roughness at a given substrate surface impact the density of potential nucleation sites, in turn influencing the density of stable nuclei.

**FIGURE 4:**
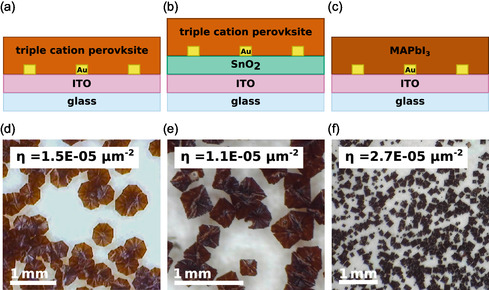
Different experimental stacks (a–c), and directly below the experimental results during recrystallization on unpatterned substrates (d–f). The different material systems result in different nucleation densities *η*.

To demonstrate the generality of the models presented in Sections 3.1 and 3.2, we choose to investigate three different combinations of perovskite composition and substrate material, shown in Figure 4a–c, which are chosen strategically to cover a large range of nucleation densities *η*. Two different perovskite compositions are studied: MAPbI_3_ and the triple cation perovskite Cs_0.1_(MA_0.17_FA_0.83_)_0.9_Pb(I_0.83_Br_0.17_)_3_. Both are deposited on ITO glass, and the triple cation perovksite is additionally studied on a SnO_2_ substrate.

To perform a seeded growth analysis within the framework of the model presented in Sections 3.1 and 3.2, the native nucleation densities *η* have to be extracted for each of the systems. Equivalent to the extraction of *η* from the phase‐field simulations in Section 3.1, we fabricate films on bare substrates without artificial nucleation centers and let them recrystallize under the same conditions later chosen for the seeded growth. Through in situ microscopic observation of the recrystallization process, the location of each new grain is recorded, thus yielding the values for *η* through a sequential image analysis further described in the methods section. Images of the films during recrystallization are shown in Figure 4d–f. The resulting values for *η* are 1.1  × 10^−5^, 1.5 × 10^−5^ and 2.7 × 10^−5^ μm^−2^. Based on these values, samples with Au seeds arranged in hexagonal patterns with different distances *D* are fabricated and analyzed. The selected seed distances *D* for each system are chosen to cover a range of $D\sqrt{\eta }$ matching the theoretical data in Figure 3. The recrystallization process is observed in situ. Sequential image analysis (further described in the Section S5) yields the total number of nuclei appearing in the observed area. From there, the number of additional grains per artificial seed is extracted, equivalent to the simulated data in Section 3.1.

**FIGURE 5:**
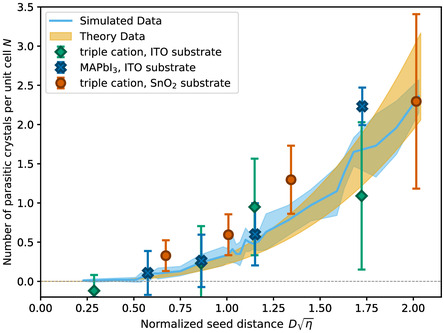
Experimental results of the number of excess grains over $D\sqrt{\eta }$ for the three material systems, along with the simulation data and analytical model curves. The experimental results match the theoretical data. The numbers shown here are the mean values derived from multiple growth processes and image analyses. The error bars correspond to the standard deviation of these values. The values result from the analysis of between 6 and 15 image sequences per data point, where each image series tracks one recrystallization process. For more details, reference the methods.

The resulting values are shown in Figure 5. The experimental results are in good agreement with both simulation and the analytical model. The error bars shown represent the standard deviations of the resulting values for all evaluated samples for this pitch.

Note that, for very low pitches, sometimes, fewer grains nucleate than seeds are present on the substrate: In contrast to the simulation, where crystals are placed at these spots initially, nucleation does not occur at all seeds simultaneously. It may occur that a grain nucleates and overgrows the neighboring seeds before nucleation has happened there. In that case, not every seed will lead to a separate grain. This occasionally gives rise to a negative number of parasitic grains at the low $D\sqrt{\eta }$.

**FIGURE 6:**
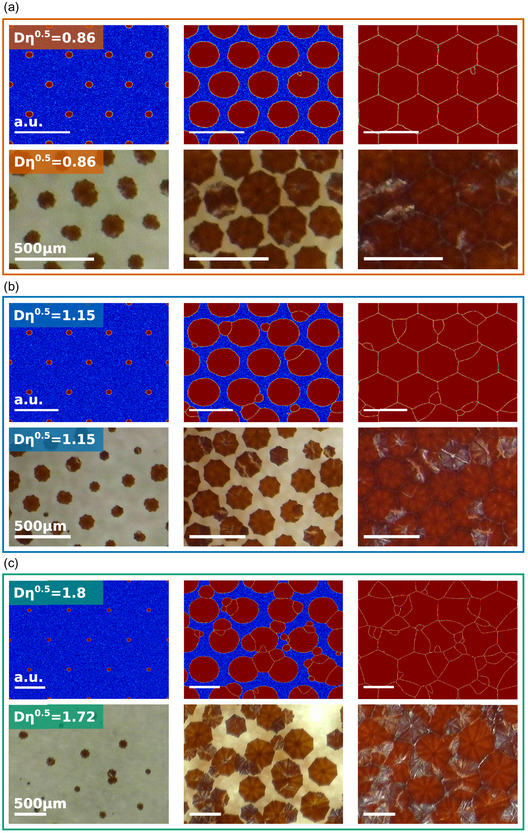
Simulated growth (top) and experiment (bottom) in direct comparison. (a) Simulation and an experiment for small *D*, where only the intended grains grow. In contrast, (b) and (c) show increasingly larger seed pitches *D* that lead to the formation of surplus grains. The temporal progression is shown from left to right. The bright spots and lines seen in the experimental images on the crystals are caused by light reflections. Since the theoretical treatment is scale‐free, the actual size of the simulations does not matter. A scale bar of arbitrary unit length is added to make the scale between the simulations in (a), (b), and (c) comparable.

Furthermore, the size of the gold seeds has to be considered carefully. If the critical radius for nucleation is smaller than the seeding spot, multiple nucleation events may happen at a single seed. This behavior can be observed for some crystals in Figure 6b. It will ultimately not impact the growth dynamic itself, and be very hard to recognize in the final film, but a “grain” started like this will consist of at least two separate crystalline domains.

Despite these differences, the model predicts the experiments remarkably well, as is visually shown in Figure 6. Both in the experiments and the simulations, the underlying hexagonal seed pattern is clearly visible. For higher pitches *D*, where $D\sqrt{\eta }\approx 1.2$ (see Figure 6a), some parasitic nucleation in between the predetermined spots occurs, while that is not the case for the lower pitches shown in Figure 6b. More examples of films during growth on substrates with differently spaced seed structures can be found in the SI in Figures S10–S12. Likewise, more examples for simulation results at different values for $D\sqrt{\eta }$ are shown in Figures S13 and S14.

## 4 Discussion

The only system specific parameter used in the model is *η* which can be used to adjust the results to a wide range of length scales and defines the possible seed arrangements. *η* exists for any material system and is not exclusive for perovskite systems; hence, the prediction should hold for any material system. Our experiments with different nucleation to growth rate ratios have already shown this flexibility for perovskites. Hence, we postulate that, as long as a continuous film is formed by nearly isotropic 2D growth of the crystals, our model is able to predict viable distances.

One can, of course, conceive several possible extensions that would open the model to even broader application (see Section S7). In its present form, the model already possesses the capability to significantly contribute to the advancement of strategic, highly optimized electronic– and optoelectronic devices. In applications and devices where the crystallographic direction and the relative position of grain boundaries are relevant, the model presented here can be used to evaluate possible layouts and to predict limitations of the system in question: For example, if crystals are to be grown across a gap between electrodes (i.e., in transistors [14, 28], or photodetectors [29]), we can predict how far apart these electrodes and the nucleation structures can be to ensure single‐grain growth as intended. This idea can be expanded to nearly all applications where crystals are grown directly onto a functional structure and where the location, crystallinity, and orientation of the crystal is of importance. That opens up a large field of research to be explored: At the moment, seeded 2D growth is frequently done from the vapor phase through epitaxy [12, 13] or vapor deposition [11]. Solution‐based methods are, however, easy to scale up and would be easily integrated in already‐established fabrication protocols. Using the understanding and the model, we propose here will aid in the systematic planning of experiments and fabrication strategies. Consequently, this work could aid in expanding on this field in a way that would be highly interesting for industrial production.

## 5 Conclusion

Millimeter‐sized grains can be created with the methylamine treatment process. By adding gold seeds onto the substrate, the spots of nucleation for new grains can be controlled, allowing for full control of size and shape of the crystal. However, parasitic nucleation may occur during this process, potentially spoiling the quality of the final film. To better understand this phenomenon, the system was simulated using a phase‐field simulation and an analytical model was developed. These models can predict the occurrence of parasitic nucleation and have been successfully validated against experiments with three different substrate‐perovskite material combinations. We find that the value of a dimensionless number, comparing the distance between the seeds and the density of crystals nucleated on a bare substrate, defines the occurrence of parasitic nucleation. The prediction of the model is unaffected by the kind of material, the length scale of the system, and the nucleation to growth rate ratio, making it very flexible in its application. Consequently, these models can be utilized for the systematic optimization of nucleation patterns in controlled 2D crystallization as demonstrated here with perovskite films.

## 6 Experimental Section

### 6.1 Sample Preparation

Samples are prepared on ITO glass substrates (Luminescence Technology Corp., 15 Ω). After four 30 min‐long steps of ultrasonication (deionized water with detergent, pure deionized water, acetone, and isopropanol), the samples are subjected to 7 min of oxygen plasma treatment.

For the samples with a SnO_2_ layer as growth interface, a SnO_2_ nanoparticle suspension (Alfa Aesar, diluted 1 mL:4.612 mL in deionized water) is spin‐coated at 3000 rpm for 30s, followed by 30 min annealing at 120°C.

Samples are then patterned with the Au seeds. After spin‐coating an adhesive layer (TI Prime, Microchemicals) at 4000 rpm for 45s and a 2 min annealing step at 120°C, a roughly 1000 nm thick photoresist (AZ MIR 701 (14 CPS), Microchemicals) layer is spin‐coated at 2000 rpm for 40s and is baked for 90s at 90°C. Illumination of the resist is performed using laser lithography (Raith, Picomaster 100, 375 nm laser source) at a dose of 250 mJcm^−2^. After illumination and a 90s bake at 120°C, the resist is developed for 60s (AZ 726 MIF, Microchemicals). After the thermal evaporation (Theva, TVA1) of 5 nm of Cr (0.1Ås^−1^) and 15 nm of Au (0.5Ås^−1^) and a lift‐off in acetone, the samples are ready for perovskite deposition.

A 1.3M precursor of the triple cation perovskite is prepared in a N_2_‐filled glovebox by dissolving 73.4 mg PbBr_2_ (Sigma–Aldrich), 507.1 mg PbI_2_ (TCI), 22.4 mg MABr (Greatcell Solar), and 172 mg FAI (Greatcell Solar) in 1 mL of a 4 : 1 mixture of DMF:DMSO and adding 53 μ L of a 389.7 mg ML^−1^ CsI (Sigma‐Aldrich) solution in DMSO per ML of the solvent mixture. The finished precursor is filtered in a 0.45 μ m PTFE filter just before perovskite deposition. For the MAPbI_3_ samples, a 1.3M precursor of MAI (Greatcell solar) and PbI_2_ (TCI) in a 9:1 DMF:DMSO mixture is used.

The perovskite layer itself is spin‐coated in N_2_ atmosphere. The triple cation precursor is dropped dynamically onto the spinning substrate at 1000 rpm. The sample is then sped up to 6000 rpm, where it remains for 25s. 20s into this faster step, 250 μ L CB anti‐solvent are dropped onto the substrate. Finally, the triple caiton perovskite layer forms during a 40 min‐long annealing step at 90°C. The MAPbI_3_ layer is spin‐coated dynamically at 3000 rpm for 20s. The layer is quenched with pressurized N_2_ for the last 10s of the program and annealed for 10 min at 100°C.

### 6.2 Methylamine Treatment Setup

**FIGURE 7:**
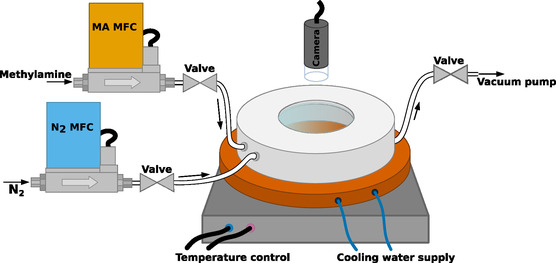
Schematic of the methylamine treatment setup. Two mass flow controllers are used to control the amount of N_2_ and MA gas let into the chamber, which can be heated and cooled from below. A vacuum pump is used to pump to a specific pressure. The process can be observed with a microscope camera through a window of optical glass from the top.

The as‐prepared perovskite samples are subjected to methylamine (MA) gas (Linde) in a specially‐built automated treatment chamber shown in Figure 7. The chamber can be heated to a specified temperature *T* with a thermoelement from below. It is equipped with two gas inlets (N_2_ and MA) and one outlet, which is connected to a vacuum pump (Welch, LVS 105 T ef + 124 184). The inlets are connected to mass flow controllers (Bürkert, MFC 8711, for N_2_ and Bronkhorst, F‐201CV‐10K‐AGD‐33‐K, for MA), allowing for control over the precise amount *N*_MA_ of gas in the chamber. The total pressure in the chamber is set by pumping to a setpoint below atmosphere pressure *p*_vac_ with the vacuum pump. Methylamine partial pressures during exposure *p*_MA, 1_ are set by letting a specified amount of gas into the chamber (Volume *V*, temperature *T*):

(5) $$\begin{array}{c} p_{\mathrm{MA},1}=\frac{N_{\mathrm{MA}}\cdot R\cdot T}{V} \end{array}$$

with the molar gas constant *R*. The total pressure at this point amounts to *p*_tot, 1_ = *p*_vac_ + *p*_MA, 1_. Recrystallization is induced by pumping the chamber down again to *p*_tot, 2_ = *p*_vac_, thus reducing the methylamine partial pressure *p*_MA, 2_ to:

(6) $$\begin{array}{c} p_{\mathrm{MA},2}=\frac{p_{\mathrm{tot},2}}{p_{\mathrm{tot},1}}\cdot p_{\mathrm{MA},1}=\frac{p_{\mathrm{vac}}}{p_{\mathrm{vac}}+p_{\mathrm{MA},1}}\cdot p_{\mathrm{MA},1} \end{array}$$

Through an optical glass window over the sample, the entire process is observed in‐situ with a microscope camera (Dino‐Lite, AF4915 ZTL).

The combinations of methylamine partial pressure *p*_MA,2_ and temperature *T* are chosen in a way that ensures slow growth of grains and strong preferred nucleation at the Au seeds. The settings for all three experiments groups are shown in Table 1.

**TABLE 1:** Recrystallization parameters for all three experimental groups. The precise combination of temperature *T*, methylamine partial pressure during recrystallization *p*_MA_ and substrate‐liquid interaction determine the nucleation densities *η* on non‐patterned substrates of each system.

Stack	*T*	*p*_MA_	*η*
Glass/ITO/MAPbI_3_	70^∘^C	195 mbar	2.7 × 10^−5^ μm^−2^
Glass/ITO/3cat	85^∘^C	230 mbar	1.5 × 10^−5^ μm^−2^
Glass/ITO/SnO_2_/3cat	80^∘^C	230 mbar	1.1 × 10^−5^ μm^−2^

### 6.3 Image Analysis and Calculation of the Number of Parasitic Crystals

During the recrystallization process, the sample is observed in situ from above. From the resulting image material, the total number of nucleated grains *N*_tot_ in a given observed area *A* can be extracted. (See the SI for a detailed description of the sequential image analysis employed to that end.) To calculate the number of parasitic grains per artificial seed at a seed pitch *D*, equivalent to the simulated data shown in the study, we normalize to the primitive hexagonal unit cell area $a=\sqrt{3}D^{2}/2$. The total number of grains per unit cell *n*_tot_ is then calculated via

(7) $$n_{\mathrm{tot}}=\frac{a}{A}\cdot N_{\mathrm{tot}}$$

If we had nucleation exclusively at the artificial seeds, we would expect exactly one grain to nucleate in each unit cell. The number of parasitic grains *N* per unit cell is thus

(8) $$N=\frac{a}{A}\cdot N_{\mathrm{tot}}-1$$

Here, the fraction $\frac{a}{A}$ corresponds to the number of unit cells that fit into the observed area *A*, which is equivalent to the number of seeds in *A*, presuming one seed per unit cell.

In Table 2, the number of analyzed images sequences are shown for each combination of pitch and material system shown in Figure 5. Note that the values shown in Figure 5 result from the mean and standard deviation of all the values of *N* extracted for one parameter combination, weighted by the areas *A* that the analyzed images show for the different image sequences.

**TABLE 2:** Number of image sequences analyzed for each combination of pitch and material system.

Stack	Pitch *D*	normalized Pitch $D\sqrt{\eta }$	Number of image sequences
Glass/ITO/SnO2/3cat	200 μm	0.672	15
Glass/ITO/SnO2/3cat	300 μm	1.008	12
Glass/ITO/SnO2/3cat	400 μm	1.344	9
Glass/ITO/SnO2/3cat	600 μm	2.016	12
Glass/ITO/SnO2/3cat	1000 μm	3.360	8
Glass/ITO/3cat	75 μm	0.287	8
Glass/ITO/3cat	225 μm	0.861	15
Glass/ITO/3cat	300 μm	1.148	16
Glass/ITO/3cat	450 μm	1.723	8
Glass/ITO/3cat	750 μm	2.871	14
Glass/ITO/MAPbI_3_	110 μm	0.575	8
Glass/ITO/MAPbI_3_	165 μm	0.863	8
Glass/ITO/MAPbI_3_	220 μm	1.50	8
Glass/ITO/MAPbI_3_	330 μm	1.73	6

Note that the number of analyzed image sequences per experimental condition varies. For the smaller pitches in particular, some experiments failed due to contamination or other uncontrolled factors, resulting in fewer analyzable sequences.

### 6.4 Determination of Phase Transition Temperatures

The phase transition points shown in the phase diagram in Figure 1b are experimentally determined for triple cation perovskite films. To achieve this, an untreated perovskite film is placed in the chamber and exposed to a defined amount of methylamine gas, corresponding to a methylamine partial pressure *p*_MA_. This is repeated at increasing temperatures until the film does not liquefy anymore upon methylamine exposure. The corresponding temperature *T* is then noted as the transition temperature for the partial pressure *p*_MA_ in question.

The precision of the resulting values is limited by the accuracy of the set temperature and that of the set methylamine partial pressure, which is denoted in Figure 1b as error bars.

## Supporting Information

Additional supporting information can be found online in the Supporting Information section. Simulation data is publicly available via https://doi.org/10.5281/zenodo.17200698. The remaining data that support the findings of this study are included in the supplementary information or available from the corresponding authors upon reasonable request.

## Author Contributions

E. S. and M. M. conceived the idea. E. S., M. M., L. S. M., O. R., and J. H. designed the project. L. S. M., O. R., and J. H. supervised the project. E. S. fabricated the samples and carried out the characterizations. M. M. performed the PF simulations and created the analytical model. E. S. and M. M. wrote the text of the manuscript. L. S. M., O. R., and J. H. revised the manuscript. All authors contributed to the discussion and commented on the manuscript.

## Funding

This study was supported by the Deutsche Forschungsgemeinschaft (SPP2196, 506698391, SPP2196, 423660474), the European Commision (H2020, 101008701/EMERGE), and the CRC1719 Chemprint (538767711).

## Conflicts of Interest

The authors declare no conflicts of interest.

## Supporting information

Supplementary Material

## Data Availability

The data that support the findings of this study are available on request from the corresponding author. The data are not publicly available due to privacy or ethical restrictions.
