# Supplementary material for: Controlling Parasitic Nucleation in Methylamine‐Treated Perovskite Films via Artificial Seeding and Phase‐Field Simulations
Source: Small Sci. 2026 May 15;6(5):e70301. doi: 10.1002/smsc.70301 (PMC13179044; doi:10.1002/smsc.70301)
Supplement: Supplementary file 1 — Supplementary Material [file SMSC-6-e70301-s001.pdf]

Supporting information of
'Controlling parasitic nucleation in methylamine-treated perovskite films via artificial seeding and phase-field simulations'

Emilia R. Schütz[a†], Martin Majewski[b†], Olivier J.J. Ronsin[b], Jens Harting[b,c*], Lukas Schmidt-Mende[a*]
a) Department of Physics, University of Konstanz, Universitätsstraße 10, Konstanz, 78464, Germany
b) Helmholtz Institute Erlangen-Nürnberg for Renewable Energy (HIERN), Forschungszentrum Jülich GmbH
c) Department of Chemical and Biological Engineering and Department of Physics, Friedrich-Alexander-Universität Erlangen-Nürnberg, Fürther Straße 248, Nürnberg
† These authors contributed equally to this work.

Email Address: j.harting@fz-juelich.de, lukas.schmidt-mende@uni-konstanz.de

S1 Large grains after MA treatment

Depending on the MA treatment conditions, substrate material, and perovskite composition, the nucleation density, which directly leads to the final grain sizes can vary wildly. Figure Figure S1 shows two exemplary images of MA treated triple cation perovskite film on SnO_2 substrates after treatment at 80 °C and a MA partial pressure of 230 mbar, which leads to the growth of very large grains than can exceed diameters of one millimeter.
Note that not only the grain diameter, but also the morphology of the film changes with treatment condition and substrate material.

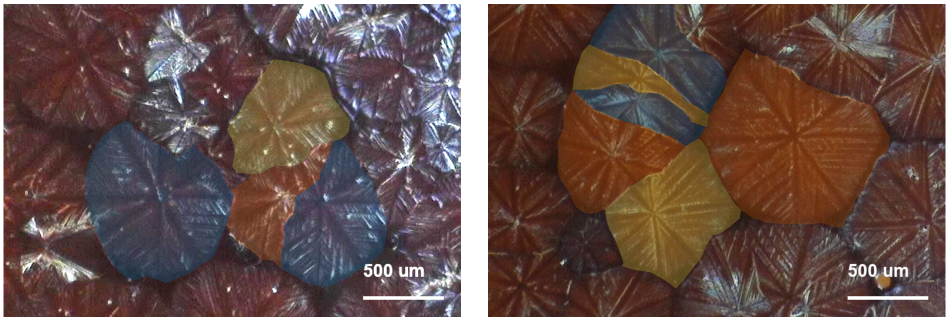

Figure S1: Microscopic images of MA treated triple cation perovskite film on SnO_2 after treatment at 80 °C and a MA partial pressure of 230 mbar. A subset of grains is highlighted using a false-color overlay to help distinguish the individual grains and indicate their size. This is one example of a combination of substrate, perovskite, and treatment conditions that leads to the growth of large grains that exceed diameters of one millimeter.

S2 Experimentally controlling nucleation during the methylamine treatment

In the phase diagram in Figure S2 a), the phase boundary between the solid and liquid states is shown based on experimentally measured data. Left of this curve, the film is liquid in equilibrium, while it is

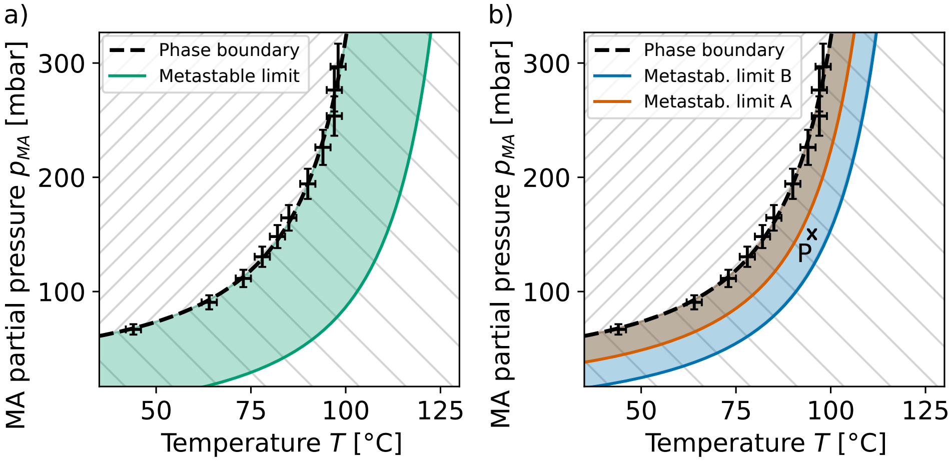

Figure S2: a) Phase diagram for the recrystallization process at hand, in case of homogeneous nucleation: beyond the phase boundary, a metastable zone exists (green), where nucleation is unlikely. b) Phase diagram and metastable zones from two different substrate materials that offer nucleation interfaces for heterogeneous nucleation. Depending on the specific interfacial energy with each material, the metastable zones will have different depths, leading to different nucleation probabilities for different materials. If crystallization is initialized at a point P, within the metastable zone for one material but not the other, nucleation can be suppressed at the surfaces of material B. Note that while the phase boundary displayed here consists of experimental data, the metastable limits are qualitative guides to the eye to illustrate the concepts involved and are not based on experimental data.

solid in equilibrium on the right side. When moving from the liquid state across the phase boundary, there is a range of temperature-pressure combinations where nucleation is unlikely to occur and the liquid state remains metastable. This metastable zone is bounded by the metastable limit curve (green line in Figure S2 a)), beyond which nucleation is likely to occur spontaneously.

The presence of the metastable zone and the position of the metastable limit can be understood from classical nucleation theory:
The formation of a nucleus is accompanied by a change in Gibbs free energy ΔG. This free energy change is comprised of a change in the volume free energy ΔG_v, and the surface free energy ΔG_s [1]:

$$\Delta G = \Delta G_v + \Delta G_s. \tag{S1}$$

The volume free energy favors nucleation, as the solid phase is more energetically favorable in the region in question. It is the driving force of nucleation and can be expressed as

$$\Delta G_v = \frac{4}{3}\pi r^3 \Delta G_v^* \tag{S2}$$

for a spherical nucleus of radius r. ΔG_v^* is the free energy difference between the liquid and solid state per unit volume. It depends on the degree of supersaturation S and the molecular volume v[1]:

$$\Delta G_v^* = -\frac{k_B \cdot T \cdot ln\,(S)}{v} \tag{S3}$$

The surface free energy ΔG_s is positive, as the formation of an interface between a solid phase and the liquid costs energy. For a spherical nucleus of radius r, it can be expressed as

$$\Delta G_s = 4\pi r^2 \gamma, \tag{S4}$$

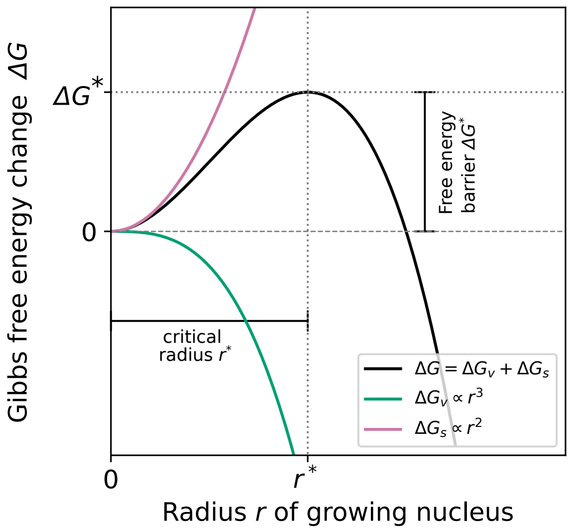

Figure S3: Gibbs free energy change ΔG for the formation of a spherical nucleus in dependence of the nucleus' radius r. The formation energy consists of a negative volume component and a positive surface component. The nucleus becomes stable above a critical radius r^*, when it has overcome the energy barrier ΔG^*.

with the surface energy per unit area γ.
In Figure S3, ΔG is shown against r. If a nucleus overcomes the critical nucleus size r^*, it becomes stable and can grow into a grain. For that to happen, the energy barrier ΔG^* has to be overcome. By finding the maximum of Equation S1, using Equation S2 to Equation S4, this energy barrier can be expressed as [1]

$$\Delta G^* = \frac{16\pi \cdot \gamma^3}{3 \cdot (\Delta G_v^*)^2} \propto \gamma^3 \propto (\Delta G_v^*)^{-2} \,. \tag{S5}$$

ΔG^* explains the existence of a metastable zone and dictates the position of the metastable limit curve. Within the metastable zone, this energy barrier prevents stable nuclei from forming. Furthermore, its dependence on ΔG_v^* directly implies an inverse dependence on the degree of supersaturation S. Moving away from the phase boundary, the supersaturation increases. Once ΔG^* is thus small enough for thermal fluctuations to overcome it, spontaneous nucleation can occur: the metastable limit is reached.
Thus far, we have only discussed homogeneous nucleation, which theoretically occurs in a medium without any impurities. The only interface, and thus the only interfacial energy of interest, is that between the liquid and solid state. However, in the thin-film system observed here, at least one other interface is possible: that with the substrate. In that case, heterogeneous nucleation occurs: nuclei form at the interface to a third material where the interfacial energy to the growing nucleus is smaller. That decreases the effective interfacial energy because the nucleus does not need to form a full spherical interface with the liquid phase: It instead forms a partial interface with (in our case) the substrate, which is energetically favorable. Due to the proportionality to γ^3 shown in Equation S5, this reduction of the effective interfacial energy decreases Gibbs free energy barrier, increasing the likelihood for nucleation.
Important to note here is that the *degree* to which the energy barrier decreases depends on the specific interfacial energies between the solid phase, the liquid and the substrate material. Different materials lead to different values of ΔG^*. In Figure S2 b), the implications for the resulting metastable zones are visualized qualitatively: Effectively, for two different materials, two different metastable limit curves arise. For the material of lower interfacial energy (in our case Au), the metastable zone is smaller, because there is a lower energy threshold to overcome for nucleation, and lower degrees of supersaturation suffice to achieve that. For the material of higher interfacial energy – in our case ITO – the metastable zone is larger.

This implies that by controlling p_{MA} and T carefully, we can choose conditions for recrystallization that lie within the metastable zone for nucleation at the ITO interface, but beyond the metastable limit for Au. We, thus, have a high probability for spontaneous nucleation *only* at the Au spots, while nucleation is suppressed everywhere else. For larger differences in interfacial energy, the gap between the two metastable limit curves is larger, making is easier to realize an effective nucleation control. Au seems to be particularly well-suited for that purpose [2], and we attribute that to a favorable interfacial energy between the growing nucleus and the Au surface.

Finally, it is to be noted that while nucleation is unlikely within the metastable zone, it is not impossible. Additionally, impurities and structural inhomogeneities such as larger spikes on the ITO substrate create additional likely nucleation points on the substrates that are not fully accounted for here. Consequently, there is always a degree of spontaneous nucleation in between the seed structures, the limits and implications of which are this paper's central focus.

S3 Simulation Model

S3.1 Free Energy

The simulation code is based on [3]. The goal is to investigate crystal nucleation on a patterned substrate. Therefore, only the transition from an amorphous state to a crystalline state is of importance. The evolution of the system is tracked by a crystalline order parameter Φ, which is zero in the amorphous state and becomes one in the crystalline state. To enable the simulation of a polycrystalline system, a so-called orientation parameter θ is recorded. This labelling field is used to distinguish the different crystals. The Gibbs free energy G is used to record the different energetic contributions:

$$G = \int_V dV \Delta G = \int_V dV G^{ls} + G^{interf}, \tag{S6}$$

where G^{ls} describes the liquid-solid phase transition and G^{interf} the interfacial contributions. G^{ls} can be written as

$$G^{ls} = \rho \left(\Phi^2 (3 - 2\Phi) L_f us (T/T_m - 1) + \Phi^2 (\Phi - 1)^2 W \right), \tag{S7}$$

with ρ the density of the material, L_{fus} is the enthalpy of fusion, T the temperature, T_m the melting temperature, and W defines the height of the energy barrier upon liquid-solid phase change. The interfacial contribution G^{interf} can be written as

$$G^{interf} = \epsilon/2 (\nabla \Phi)^2 + (3 - 2\Phi)\Phi^2 \pi \epsilon_g \delta(\nabla \theta)/2, \tag{S8}$$

where ϵ defines the contribution to the surface energy arising from the gradients in the crystalline order parameter, ϵ_g for the orientation parameter, and $\delta(\nabla\theta)$ is defined to be one if a difference in orientation is present and zero elsewhere.

S3.2 Dynamic Equation

The evolution of the system is given by the stochastic Allen-Cahn equation:

$$\frac{\partial \Phi}{\partial t} = -\frac{\nu_0}{RT} M \left(\frac{\partial \Delta G}{\partial \Phi} - \nabla \frac{\partial \Delta G}{\partial (\nabla \Phi)} \right) + d(\Phi, d_\chi, w_\chi, c_\chi)\chi, \tag{S9}$$

where ν_0 is the molar volume, R the gas constant, M the mobility, d an interpolating function and χ fluctuations with zero mean and a second moment of

$$\langle \chi(x,t), \chi(x',t') \rangle = \frac{2\nu_o}{N_a} M \delta(t - t') \delta(x - x'), \tag{S10}$$

with N_a being the Avogadro number. The interpolating function can be written as

$$\log(d(d_\chi, w_\chi, c_\chi) = 0.5 \log(d_\chi) \left(1 + \tanh(w_\chi(\Phi - c_\chi)) \right), \tag{S11}$$

whose shape is defined by w_χ, d_χ, c_χ. The evolution of the orientation field is given as follows: If the crystalline order parameter rises above a certain threshold t_Φ, then we assign the orientation parameter of the nearest crystal or a new one, if no crystal is present nearby.

S3.3 Simulation Setup

The top view of the experiment is simulated. The two-dimensional simulation box has periodic boundary conditions. Sixteen crystals are initially placed in a triangular lattice. The setup is shown in Figure S4. The input parameters are listed in Table S1.

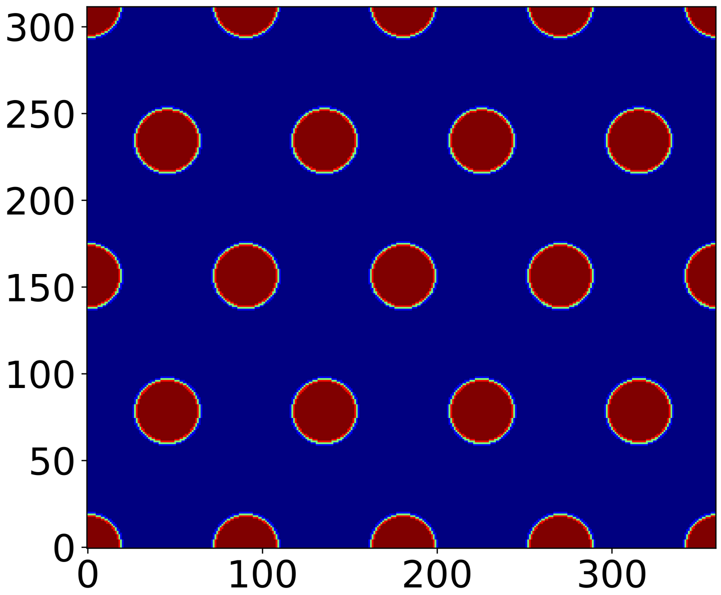

Figure S4: Simulation setup for one pitch. The amorphous phase is shown in blue and the crystalline phase in red.

S3.4 Simulation Parameters

Table S1: Parameters of the simulations

Parameters	Full Name	Value	Unit
dx, dy	Grid spacing	1	nm
nx, ny	Grid size	varied	-
T	Temperature	460	K
ρ	Density	1000	kg/m^3
m	Molar mass	0.1	kg/mol
ν_0	Molar volume of the Flory Huggins lattice site	10^{-4}	m^3/mol
T_m	Melting temperature	600	K
L_{fus}	Heat of fusion	75789	J/kg
W	Energy barrier upon crystallization	142105	J/kg
P_0	Reference pressure	10^5	Pa
ϵ_c	Surface tension parameters for order parameter gradient	$1.4 \cdot 10^{-5}$	$(J/m)^{0.5}$
M_c	Allen Cahn mobility coefficient for the crystalline phase	48	s^{-1}
d_χ, c_χ, w_χ	Amplitude, center and width of the penalty function for the fluctuations	10^{-2}, 0.85, 15	-

S3.5 Nucleation to growth rate ratio

The influence of the nucleation-to-growth rate ratio is investigated. To this end, two sets of simulations are performed, varying the seed distance and the nucleation-to-growth rate ratio. As shown in Figure S5, this ratio leads to changes within the statistical uncertainties. The scaling with the nucleation density η on the x-axis is the reason for this behavior.

S3.6 Initial crystal size

The influence of the initial crystal sizes is investigated. To this end, two sets of simulations are conducted, varying the distances between the crystals and the crystal sizes. As shown in Figure S6, this size does not matter. The reason is as follows: the distance plotted on the x-axis represents the minimal distance between the surfaces of the crystals, which compensates for the shift in the graph resulting from varied initial crystal sizes.

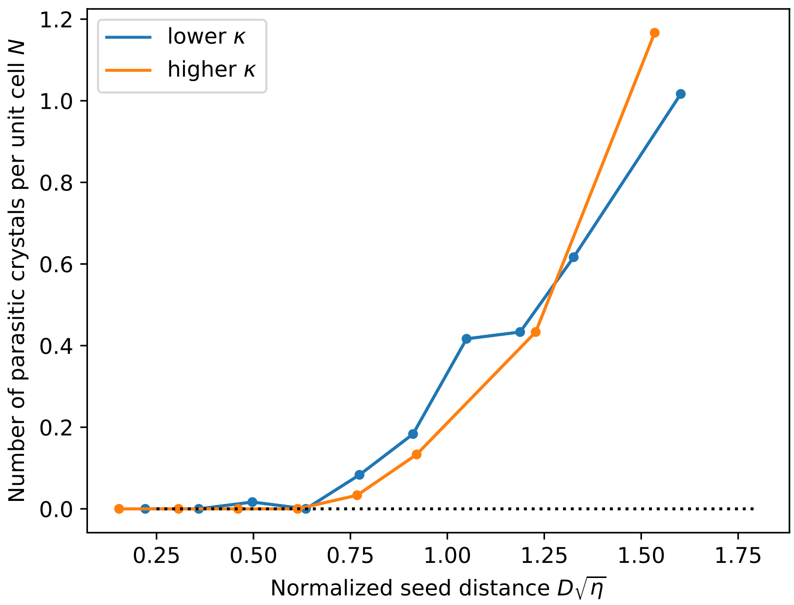

Figure S5: Evaluation of the number of parasitic grains for two different nucleation rates. Since the statistical errors for this process are rather large (see Figure 2, main text), the curves are equivalent, and therefore the ratio between growth and nucleation does not alter the result.

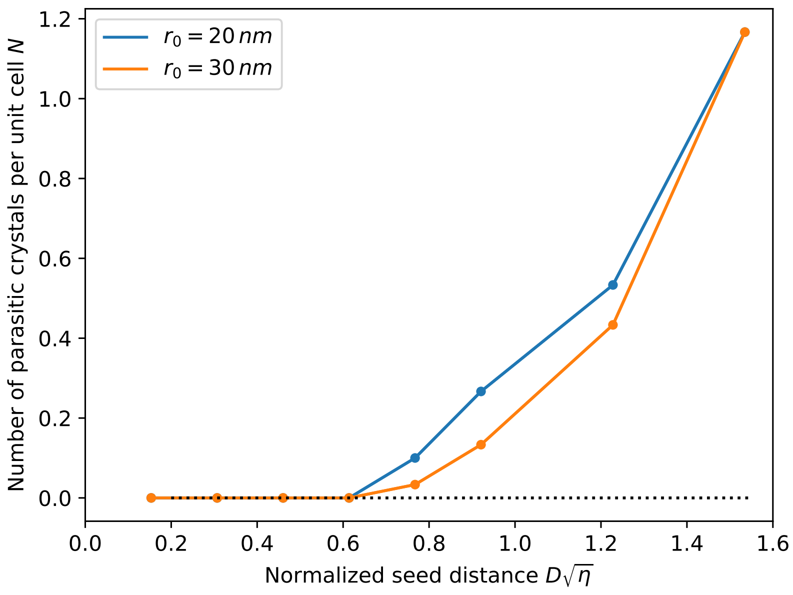

Figure S6: Evaluation of the number of parasitic grains for two different initial crystal sizes. Since the statistical errors for this process are rather large (see Figure 2, main text), the curves are equivalent. Therefore, the exact initial crystal sizes do not alter the result as long as the size of the crystals is similar to the seed distances.

S4 Analytical model

The experiment utilizes a triangular lattice of gold seeds, where the crystals preferentially nucleate. The dependence of the number of parasitic crystals on the pitch is investigated both numerically and analytically. For the analytical model, the crystal nucleation rate, crystal growth rate, and lag time of nucleation are required as input. These values can be evaluated from the simulations.

The setup is as follows: The area between three seeds is analyzed. The crystal center-to-center distance is called D. Therefore, the total area is

$$A = D^2/2, \tag{S12}$$

The time until the crystals impinge on each other is

$$t < D/(2v_g). \tag{S13}$$

It is assumed that crystals can nucleate anywhere within the amorphous phase with a rate of κ. In the simulation, crystals are placed on a triangular lattice. Since the size of the crystals does not matter (see Figure S6), we set the initial size of the crystals to zero in the model, hence:

$$A_c(t) = (v_g \cdot t)^2 \cdot \pi/2 \tag{S14}$$

Each crystal is part of 6 unit cells in an infinite lattice. Therefore, each of the three circles in the triangle contributes 1/6 of its area.

Additionally, there is a nucleation induction time until the first crystal appears. This time is called t_0. The total amount of parasitic grains N is therefore

$$N = \int_{t_0}^{D/2v_g} dt(A - A(t))\kappa. \tag{S15}$$

Integration yields:

$$N = \frac{\kappa D^3}{4v_g}\left(1 - \frac{\pi}{12}\right) + \frac{\kappa D^2 t_0}{2}\left(\frac{t_0^2 v_g^2}{3} - 1\right). \tag{S16}$$

The simplifications used are:

- A lower limit is set for $t = D/v_g 2$, where the area in between the three impinging circles is still not crystalline.
- An upper limit is approximated with $t = 1.15D/v_g 2$, where all the material of the system is crystallized due to the growing seeds.
- The area covered by nucleated crystals is neglected, and hence, for long seed distances, it overestimates the number of parasitic grains.

S4.1 Analytical model - parameters

The input parameters to be set are the nucleation rate κ, the crystal growth rate v_g, and the nucleation induction time t_0. The crystal growth rate v_g can be measured by simulating one growing crystal. We observe a value of $v_g = 142\ nm/s$ for the simulation parameters. For the nucleation rate κ and nucleation induction time t_0, we use the same simulations, where η has been evaluated. We obtain a nucleation induction time of $t_0 = 0.1s$ and a nucleation rate of $\kappa = 2.1 \cdot 10^{-5} nm^{-2} s^{-1}$. The initial crystal size is set to $0\ nm$, but this value is not relevant for the results, as shown in Figure S6.

S4.1.1 Evaluation of the nucleation rate and nucleation induction time

The nucleation rate and the nucleation induction time are evaluated from a simulation with no initially placed crystals. All further parameters are as stated above. Within the simulation, the nucleation induction time is defined as the time interval from t = 0 to the time at which the first crystal reaches the size of

a critical germ. The number of crystals (larger than the critical germ size) and the amount of amorphous area are tracked. Its time derivative is scaled by the (time-dependent) amount of amorphous material to obtain the nucleation rate. This rate is averaged over time to obtain a constant value.

S4.1.2 Crystal growth rate

The crystal growth rate is measured in a separate simulation, where a single crystal is placed within a box. Fluctuations increase the growth rate compared to the theory (simulations without fluctuations). Therefore, they are turned on. The area of the placed crystal is tracked until it impinges on newly nucleated crystals, and an equivalent radius of the circle is calculated. The growth rate is the time derivative of the radius's evolution.

S4.2 Calculating the nucleation density

According to the Johnson-Mehl-Avrami-Kolmogorow (JMAK) theory [4], the amorphous phase $A(t)$ in a system with homogeneously crystallizing crystals in 2D evolves as

$$\frac{A(t)}{A_{tot}} = \exp\left(-\frac{1}{3}\pi v_g^2 \kappa t^3\right), \tag{S17}$$

where A_{tot} is the total area of the system, v_g the growth rate of the crystals and κ the nucleation rate. The nucleation density η can be calculated as

$$\eta = \frac{N}{A_{tot}} = \int_0^\infty dt A(t)\kappa, \tag{S18}$$

where N is the total number of crystals in the system. Integrating numerically (with the nucleation and growth rate extracted from the simulation) yields $\eta_{num} \approx 2.7 \cdot 10^{13}\, m^{-2}$. The value measured in the simulation ($\eta_{sim} \approx 2.2 \cdot 10^{13}\, m^{-2}$) is substantially smaller. This is not unexpected, as JMAK considers only pairwise impingement and therefore overestimates the available area. Hence, we will use the simulated value for higher accuracy.

S5 Sequential image analysis for the detection of the number of nucleating grains

The number of nucleation points per area is established via the image analysis of a sequence of microscope images acquired sequentially during grain growth by a camera placed above the sample. Each image is first analyzed to determine the centroid points of all grains or grain clusters appearing on the sample. Through a comparison of all centroid points appearing on one image to those on the next image, the true nucleation sites are then identified.

In Figure S7, the image processing done to each image is shown. The original image (Figure S7 a)) is first converted to greyscale values (Figure S7 b)). The resulting intensity values are then subjected to thresholding to separate the light background from the dark grains, resulting in a binary image (Figure S7 c)). This binary image will usually still contain some artifacts: ideally, each grain will be a solid area of 'true' color values at this point, and the background will be solidly 'false'. Due to intensity variations on the grains and on the substrate, i.e. due to light reflections, some pixels on the grains will appear too light to be correctly identified as 'true' and vice versa. To eliminate these artifacts, the image is opened, then closed at this point. Opening is a technique used in morphological processing to eliminate small areas below a critical radius, getting rid of noise on the substrate. Closing is the inverse and will fill in missing pixels in the otherwise solid grain regions. A resulting binary image is shown in Figure S7 d). In this image, connected regions of true pixels can be identified (Figure S7 e)). Their size and, crucially, their centroid location can be extracted. The centroid locations extracted this way from the original image are shown overlayed in Figure S7 f).

Analyzing the entire image sequence of grain growth, from the appearance of first nuclei until the substrate is fully covered, the location and number of true nucleation sites can now be determined. As clearly

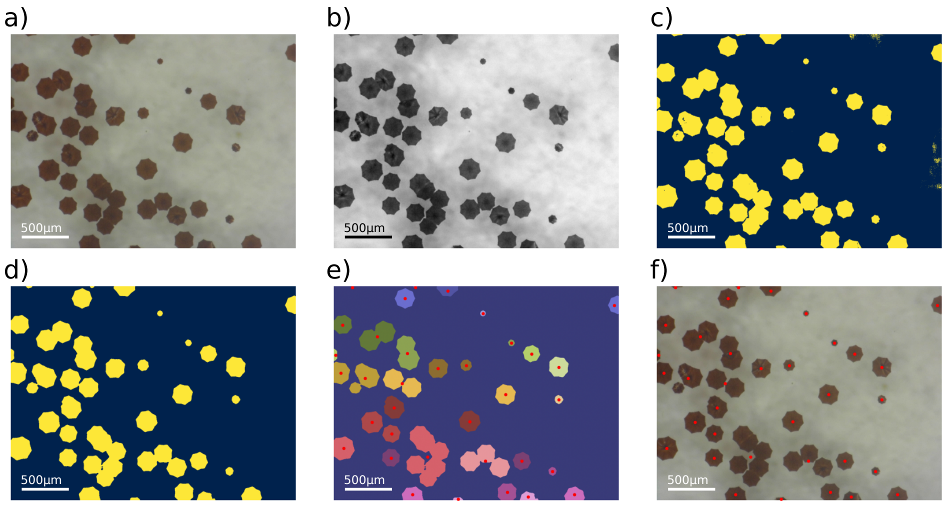

Figure S7: Example of the image processing done to determine the centroid points of all grains appearing in one in-situ image. The original image (a) is converted to greyscale (b). The resulting intensities are thresholded (c) to get a binary image. Opening and closing algorithms are used to get rid of artifacts occurring during thresholding (d). These binary regions are then analyzed (e) to determine the centroid points of each visible grain or grain cluster. The resulting centroids coincide with the grain centers, .i.e, with the nucleation point (f), provided the grain is relatively small and has not yet grown too far or fused with a neighboring grain.

visible in Figure S8 a)-c), the processing of individual images yields the position of the centroid of each grain or grain cluster at that time. In the initial phase of grain growth, this point coincides with the nucleation site, as the grain is still small, grows relatively uniformly in all directions and has not yet fused with any neighboring grains. As the substrate is covered more and more fully, grains meet and fuse into larger regions, whose centroid locations do not correspond to any nucleation points anymore.
To identify the true nucleation sites, the centroid positions of each image are compared to those of the next sequentially. If a new centroid appears from one image to the next, the reason has to be either a newly nucleated grain, in which case this point can be noted as a nucleation center, or the meeting of two preexisting grains, which means the new centroid is to be ignored. Conversely, if a centroid disappears from one image to the next, we have to assume the corresponding grain has joined with a neighboring one. Upon the joining of two grains with areas A and B and centroids (x_A, y_A) and (x_B, y_B), the new centroid coordinates will be given by:

$$x_{new} = \frac{x_A \cdot A + x_B \cdot B}{A + B}, \qquad y_{new} = \frac{y_A \cdot A + y_B \cdot B}{A + B} \tag{S19}$$

Therefore, to distinguish between 'real' new nucleation sites and 'false' new joined centroids, we can compare the coordinates of all disappeared old centroids and the areas of the corresponding regions to the coordinates of all new centroids. If two disappeared centroids can be attributed to a new one as described in Equation S19, the new coordinates can be discarded. Doing this for all images recorded, the true nucleation centers can thus be identified. To visualize this, Figure S9 shows the final result: all nucleation centers that are recorded over the span of the recrystallization process are marked in pictures at different times.

In practice, the image analysis is implemented in Python, using the *skimage* package for image processing. This includes the thresholding operations, the opening and closing algorithms, and the identification of morphological regions and their properties.
The full script can be found under `https://github.com/erschuetz/MATreatmentImageAnalysis.git`.

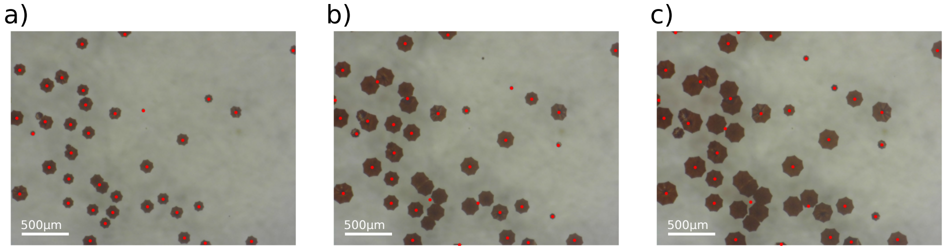

Figure S8: Series of images as they are recorded during grain growth with the identified centroid points. While some grains have not touched yet in (a) and generate their own centroid points, they will eventually meet as demonstrated in (b) and (c), resulting in joined centroid points that are not associated with an actual nucleation site.

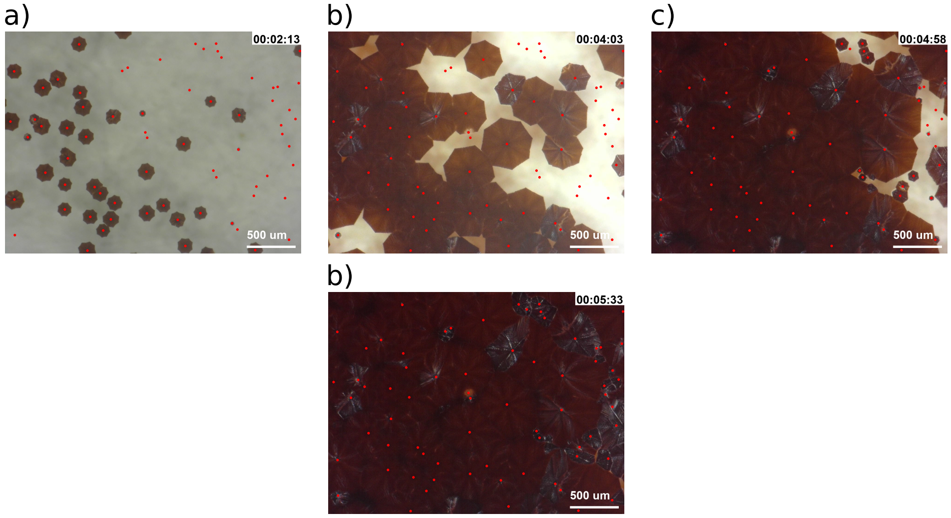

Figure S9: Series of images as they are recorded during grain growth with the identified true grain centers on the same sample. Time progresses from a) to d). The algorithm tracks the appearance of new grains and records their nucleation points.

S6 Examples of the experimental growth process and simulation data for different values of $D\sqrt{\eta}$

To illustrate the range of structures grown experimentally for different seed pitches D in the different material systems, exemplary image sequences are shown in Figure S10, Figure S11, and Figure S12 for the ITO/triple cation, ITO/$MAPbI_3$, and SnO_2/triple cation stacks, respectively. Two different image sequences for the same pitch D and under identical conditions are shown. a) and b) are films with a large pitch D with significant parasitic recrystallization, while b) and c) present the results for a smaller value of D. The exact values for D and $D\sqrt{\eta}$ are detailed in the figure captions.
Likewise, to demonstrate the simulation data for different pitches D, exemplary simulation results for various values of $D\sqrt{\eta}$ are shown in Figure S13 and Figure S14. The Figures shown are sorted for increasing values of $D\sqrt{\eta}$.

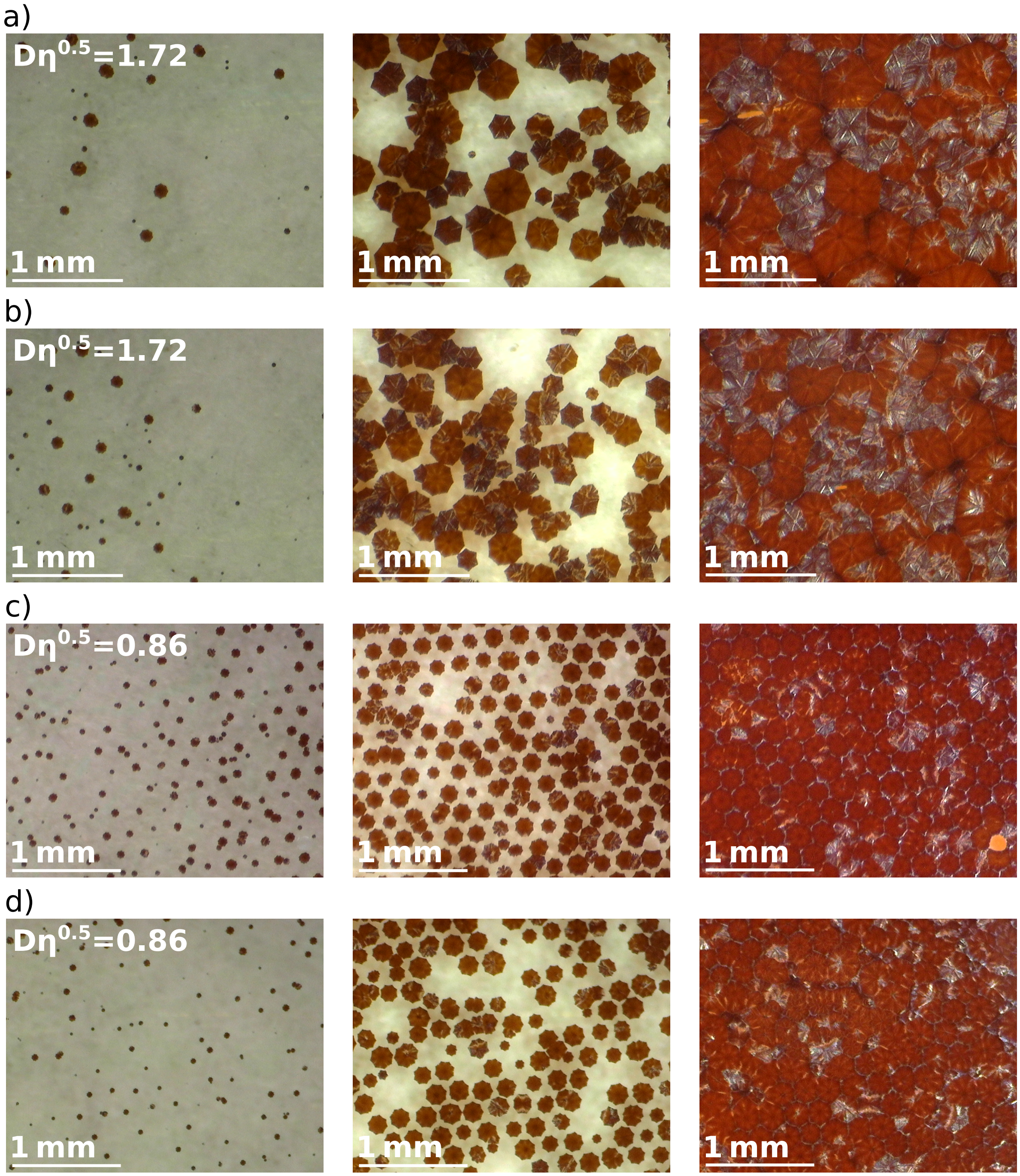

Figure S10: Image sequences of triple cation perovskite films on ITO substrates during recrystallization at at pitch of a)-b) 450 µm and c)-d) 220 µm, corresponding to values of 1.72 and 0.86 for $D\sqrt{\eta}$, respectively. Time progresses from left to right.

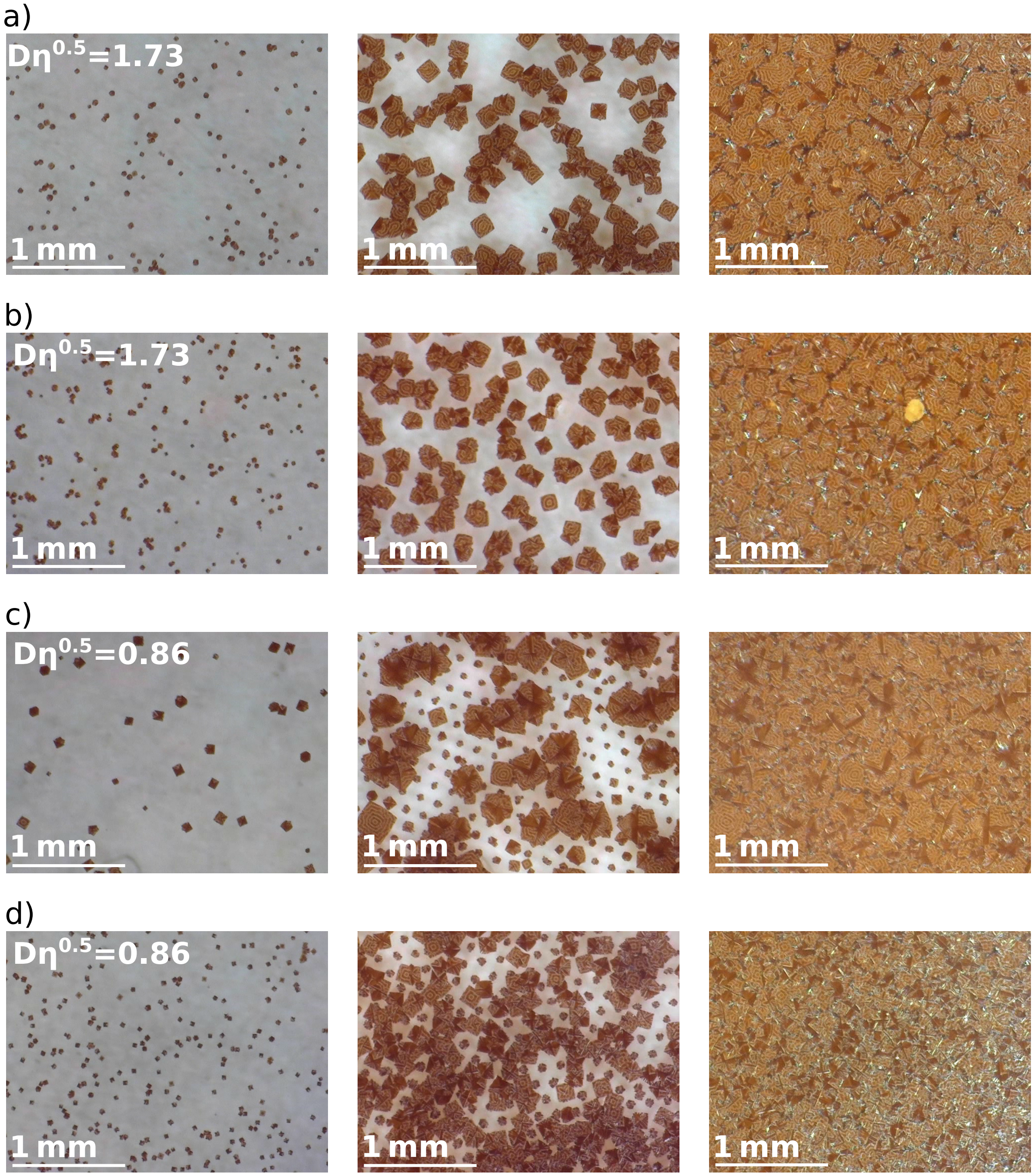

Figure S11: Image sequences of $MAPbI_3$ perovskite films on ITO substrates during recrystallization at at pitch of a)-b) 330 µm and c)-d) 165 µm, corresponding to values of 1.73 and 0.86 for $D\sqrt{\eta}$, respectively. Time progresses from left to right.

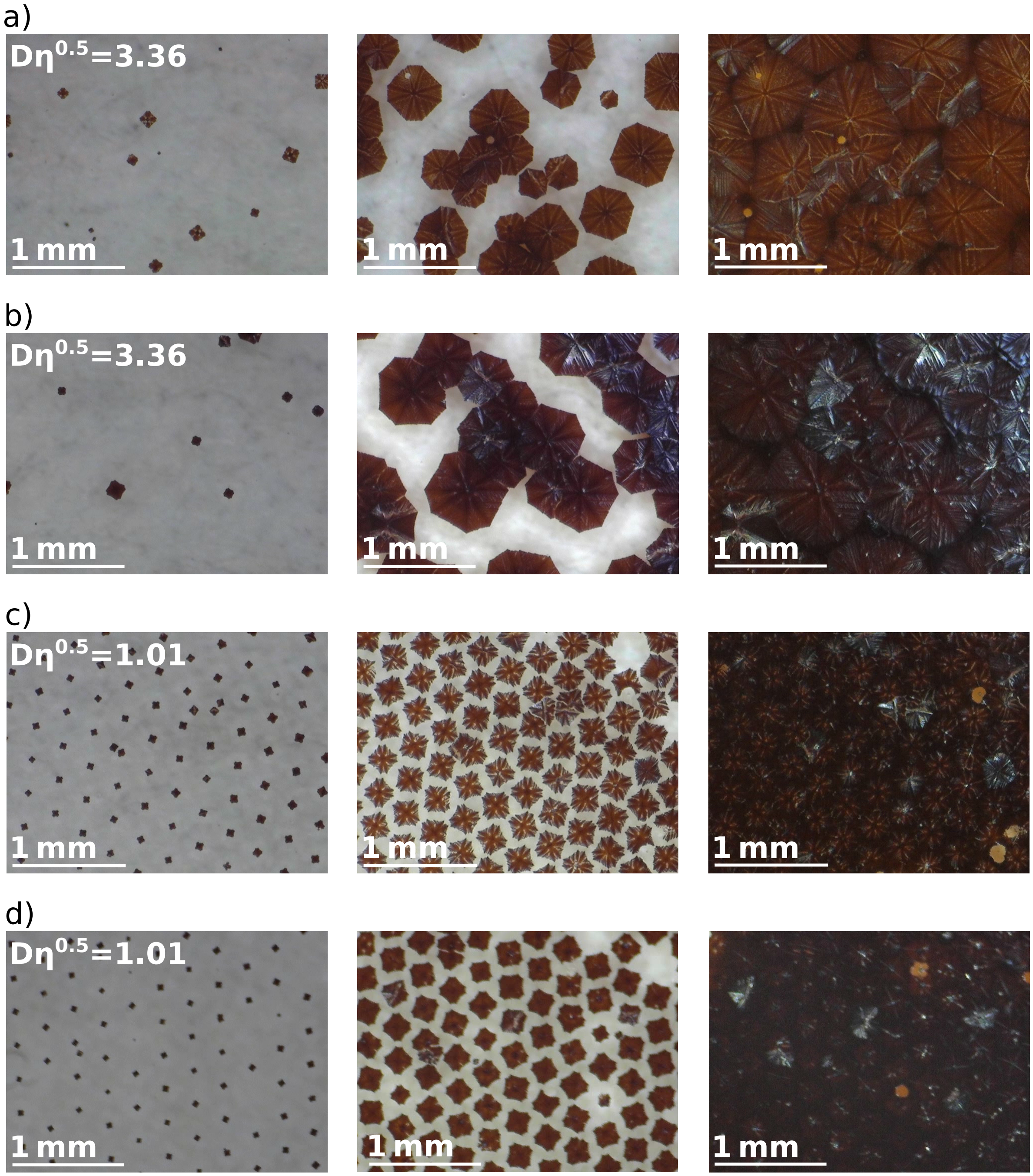

Figure S12: Image sequences of triple cation perovskite films on SnO_2 substrates during recrystallization at at pitch of a)-b) 1000 µm and c)-d) 300 µm, corresponding to values of 3.36 and 1.01 for $D\sqrt{\eta}$, respectively. Time progresses from left to right.

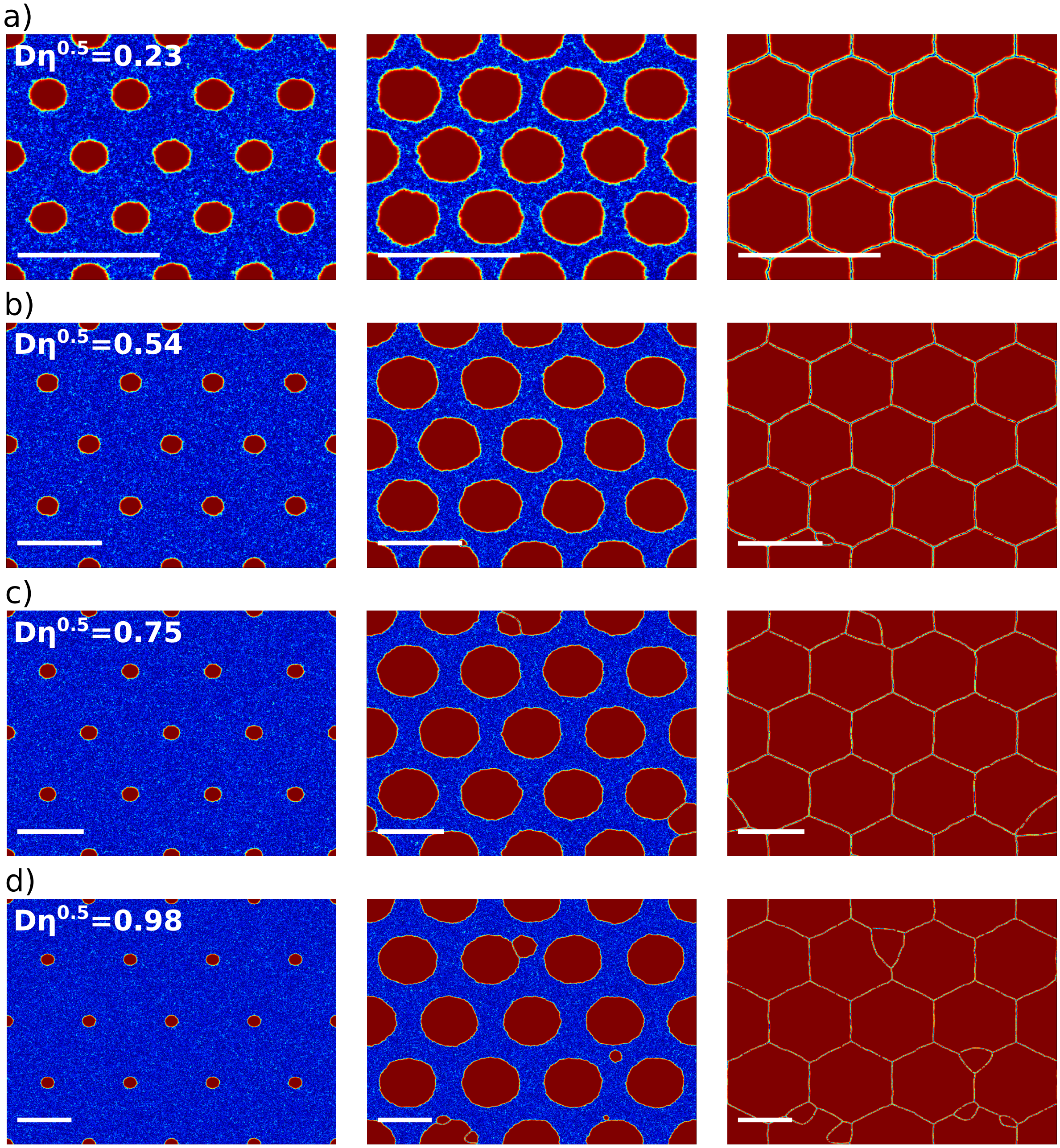

Figure S13: Exemplary simulation results for values of $D\sqrt{\eta}$ between 0.23 and 0.98. Time progresses from left to right, showing the expansion of the crystalline phase (brown) starting from the seed points. The scale bar indicates the same arbitrary length in Figures S13 and S14.

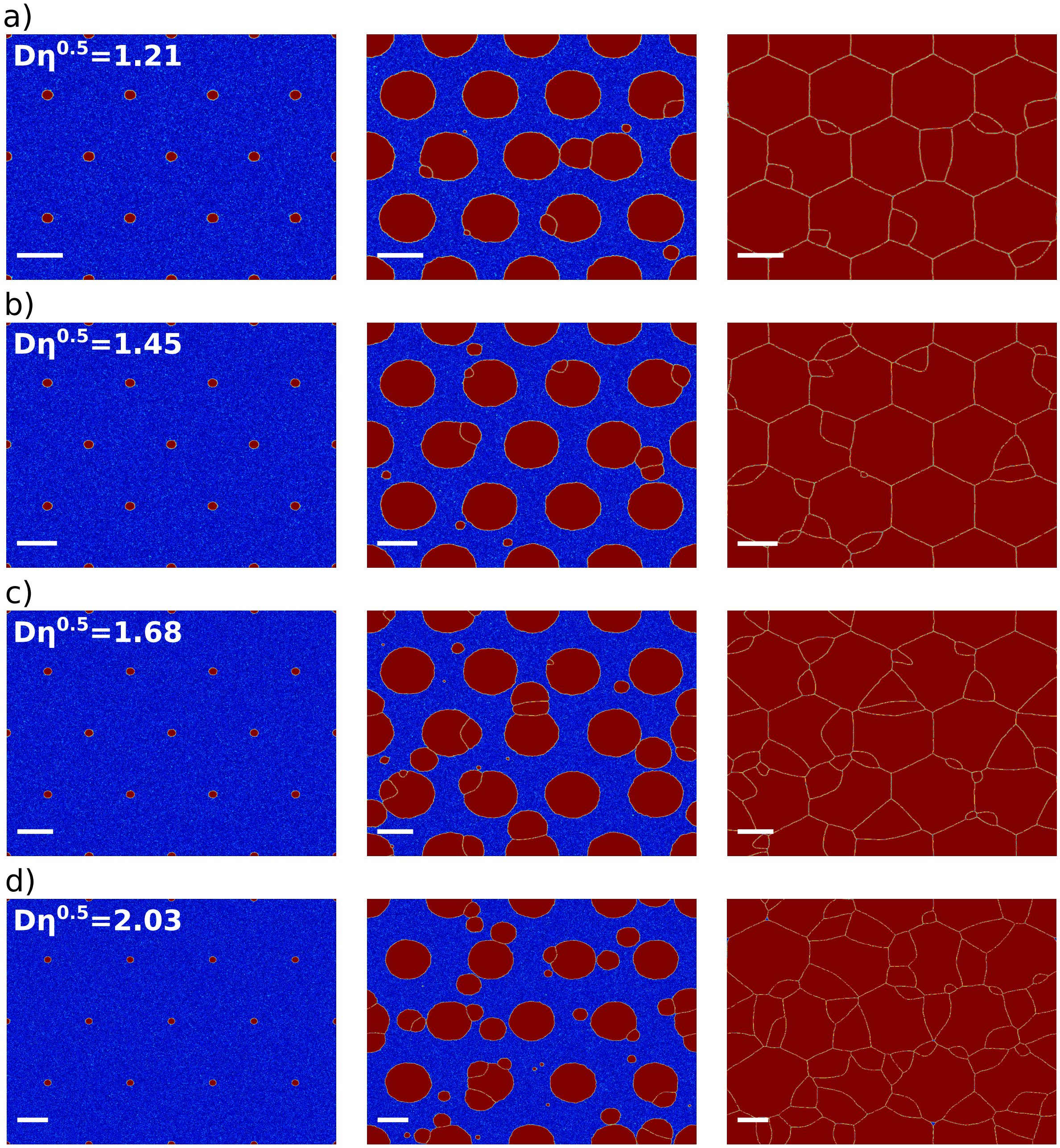

Figure S14: Exemplary simulation results for values of $D\sqrt{\eta}$ between 1.21 and 2.03. Time progresses from left to right, showing the expansion of the crystalline phase (brown) starting from the seed points. The scale bar indicates the same arbitrary length in Figures S13 and S14.

S7 Model Extensions

First, the shape of the patterning seeds may be changed, to reflect and investigate real achievable seeds and patterns. Second, the model could be extended to anisotropic growth mechanisms. As is visible in Fig. 6 (main text), the perovskite films under methylamine atmosphere exhibit preferred growth directions in accordance to the forming crystal lattice. Though this anisotropy is not sufficiently strong in this case to impact the findings from the model, an according extension of the model would be highly interesting for more anisotropic materials.

The analytic model and simulations could also be modified to include a third dimension. Under certain circumstances, there are gaps forming in between the final crystals in the experiment [5]. Especially for tight patterns or very slow growth speeds, material may run out before the next grain is met. The origin of this phenomenon would be an interesting starting point for further investigations. To reproduce this phenomenon we expect the simulations have to be more complex by including the third dimension. Additionally, a second material (air) may be necessary to allow for a free surface resulting in pinholes. Furthermore, the simulations and model could be modified to describe a three dimensional system instead of the thin-film based 2D system presented here.

Experimentally, patterning systematically in three dimensions would be a challenging endeavor. While one could think of specific scenarios, i.e. inducing nucleation with a light intensity pattern, or mounting nucleation seeds on vertical structures, the experimental effort would be considerable. However, introducing artificial seeds as suspended particles during the fabrication of nanoparticles [6] or thin films [7] is established practice. Such a suspension acts as a statistical distribution of seeds with average distances, and as such could likely be modeled by a similar scheme.

References

[1] J. W. Mullin, *Crystallisation*, Butterworths, London, 2 edition, **1972**.

[2] T. Geske, J. Li, M. Worden, X. Shan, M. Chen, S. G. R. Bade, Z. Yu, *Advanced Functional Materials* **2017**, *27*, 40 1702180.

[3] O. J. J. Ronsin, J. Harting, *Advanced Theory and Simulations* **2022**, *5*, 10 2200286.

[4] C. D. Van Siclen, *Physical Review B* **1996**, *54*, 17 11845.

[5] A. Günzler, E. Bermúdez-Ureña, L. A. Muscarella, M. Ochoa, E. Ochoa-Martínez, B. Ehrler, M. Saliba, U. Steiner, *ACS Applied Materials & Interfaces* **2021**, *13*, 5 6854.

[6] M.-Q. He, Y. Ai, W. Hu, L. Guan, M. Ding, Q. Liang, *Advanced Materials* **2023**, *35*, 46 2211915.

[7] Y. Zhao, H. Tan, H. Yuan, Z. Yang, J. Z. Fan, J. Kim, O. Voznyy, X. Gong, L. N. Quan, C. S. Tan, J. Hofkens, D. Yu, Q. Zhao, E. H. Sargent, *Nature Communications* **2018**, *9*, 1 1607.
